# Physiological Evidence for a Midline Spatial Channel in Human Auditory Cortex

**DOI:** 10.1007/s10162-016-0571-y

**Published:** 2016-05-10

**Authors:** Paul M. Briley, Adele M. Goman, A. Quentin Summerfield

**Affiliations:** Department of Psychology, University of York, York, YO10 5DD UK; Hull York Medical School, University of York, York, YO10 5DD UK

**Keywords:** sound localization, spatial tuning, opponent process, electroencephalography, EEG, auditory system

## Abstract

Studies with humans and other mammals have provided support for a two-channel representation of horizontal (“azimuthal”) space in the auditory system. In this representation, location-sensitive neurons contribute activity to one of two broadly tuned channels whose responses are compared to derive an estimate of sound-source location. One channel is maximally responsive to sounds towards the left and the other to sounds towards the right. However, recent psychophysical studies of humans, and physiological studies of other mammals, point to the presence of an additional channel, maximally responsive to the midline. In this study, we used electroencephalography to seek physiological evidence for such a midline channel in humans. We measured neural responses to probe stimuli presented from straight ahead (0 °) or towards the right (+30 ° or +90 °). Probes were preceded by adapter stimuli to temporarily suppress channel activity. Adapters came from 0 ° or alternated between left and right (−30 ° and +30 ° or −90 ° and +90 °). For the +90 ° probe, to which the right-tuned channel would respond most strongly, both accounts predict greatest adaptation when the adapters are at ±90 °. For the 0 ° probe, the two-channel account predicts greatest adaptation from the ±90 ° adapters, while the three-channel account predicts greatest adaptation when the adapters are at 0 ° because these adapters stimulate the midline-tuned channel which responds most strongly to the 0 ° probe. The results were consistent with the three-channel account. In addition, a computational implementation of the three-channel account fitted the probe response sizes well, explaining 93 % of the variance about the mean, whereas a two-channel implementation produced a poor fit and explained only 61 % of the variance.

## **INTRODUCTION**

Sound *frequency* is represented topographically in auditory cortex and subcortex where many neurons respond to narrow ranges of frequencies, with adjacent neurons responding to similar ranges and distant neurons responding to different ranges (Gulick et al. 1989). This arrangement is much like the representation of retinal location in the visual system (Hubel 1995). For sound *location*, in comparison, physiological studies with humans (Salminen et al. 2009, 2010; Magezi and Krumbholz 2010; Briley et al. 2013; Briley and Summerfield 2014; Stecker et al. 2015; Trapeau and Schönwiesner 2015; McLaughlin et al. 2016) and anaesthetized mammals (McAlpine et al. 2001; Stecker et al. 2005a; Werner-Reiss and Groh 2008) have suggested that spatial coding in the frontal horizontal (“azimuthal”) plane is achieved by just two broadly tuned groups of neurons, one maximally responsive to left auditory space and the other to right auditory space (Boehnke and Phillips 1999). Both groups are present in each auditory cortex, though the contralateral channel is likely to be dominant (see Briley et al. 2016, for recent physiological results from humans). In this account, sound-source location is coded by the balance of activity in the two channels, as opposed to the most active of the array of channels in a topographic representation. Analogous opponent processing underlies the perception of colour in the visual system (DeValois and DeValois 1993).

Phillips and colleagues have provided extensive evidence for the two-channel opponent-process model from human psychophysical studies (Boehnke and Phillips 1999; Phillips et al. 2003, 2006; Phillips and Hall 2005; Vigneault-MacLean et al. 2007). For example, Vigneault-MacLean et al. (2007) found that presenting a lateralized “adapter” stimulus biased the perception of a subsequent “probe” stimulus towards the opposite side. This effect occurred across a wide range of probe locations in the same hemifield as the adapter, as well as probe locations close to the midline in the opposite hemifield. Such a wide-ranging effect would be expected under a two-channel model in which the channels have broad tuning curves that just span the midline. The lateralized adapter stimulus would suppress activity from the channel responsive to its hemifield (the ipsilateral hemifield) more than it would the channel responsive to the opposite (contralateral) hemifield. As a result, the balance of activity in the two channels would tend to favour the contralateral channel, signalling a probe location further towards the contralateral hemifield than actually presented.

Recently, however, Phillips and colleagues have argued for a modification to the two-channel model to incorporate a third channel maximally responsive to the midline (Dingle et al. 2010, 2012). This proposition is consistent with recent animal physiological studies showing evidence for a substantial number of midline-tuned neurons in awake, as opposed to anaesthetized, mammals (Lee and Middlebrooks 2011, 2013; Zhou and Wang 2012). Dingle et al. altered their previous psychophysical paradigm by presenting adapter stimuli at the midline or by alternating adapter stimuli between extreme left and right azimuthal locations. They found that the alternating adapters shifted the perceived probe location towards the midline, whilst the midline adapter shifted the perceived probe location away from the midline. Dingle et al. argued that a two-channel model would predict no change in the perceived probe location in either of these adapter conditions. This is because the adaptation is always symmetric—it would leave the balance of activity in the two channels unaffected. In contrast, a three-channel model would predict the observed results. The left and right adapters would suppress the activity of the left and right channels more than that of the midline channel, meaning that the balance of activity between the channels to a subsequent probe would favour the midline channel. The midline adapter would shift perceived probe location away from the midline, since this adapter would particularly suppress the activity of the midline channel and the balance of activity to the probe would then favour the left/right channels.

In the current study, we sought physiological evidence in humans for a midline-tuned channel. Conclusions from animal physiological studies may not be fully applicable to humans given differences in head size and frequency range of hearing. We recorded neural responses using electroencephalography (EEG) to probes located at the midline (0 °) or to the right (+30 ° or +90 °); probes were preceded by adapter stimuli that occurred at the midline or alternated between −30 ° and +30 ° or between −90 ° and +90 °. In line with previous human physiological studies investigating spatial representation with adaptation (Salminen et al. 2009, 2010; Magezi and Krumbholz 2010; Briley et al. 2013; Briley and Summerfield 2014), we assumed that the EEG response to a probe represents a summation of the outputs of the different spatial channels to the probe location. Note, therefore, that the EEG responses are expected to differ from the psychophysical responses, which are assumed to depend on the output of the process which compares the channels. In our study, the two-channel account predicts smallest probe responses following the ±90 ° adapters, since these adapters would suppress the activities of the left and right channels the most. The three-channel account makes the same prediction for the +90 ° probe. However, the midline channel responds most strongly to the 0 ° probe; therefore, the midline (0 °) adapters are predicted to have greatest suppressive effect on the response to the 0 ° probe. Predictions for the +30 ° probe depend on the shapes of the three channels. We evaluated these predictions first by comparing the electrophysiological responses to the probe stimuli and second by modelling the probe responses computationally.

## **METHODS**

### Participants

Sixteen young adults (mean age ± SD, 22.8 ± 3.5, six males) participated. None reported a history of audiological or neurological disease, and all had pure-tone hearing thresholds at, or more favourable than, 25 dB HL at octave frequencies between 250 and 4000 Hz, inclusive. The study was approved by the Research Ethics Committee of the Department of Psychology of the University of York and was conducted in accordance with the Declaration of Helsinki of the World Medical Association. Participants gave informed written consent.

### Procedure

EEG measurements were made in a single-walled IAC audiology test room, located in a larger sound-treated enclosure. Participants sat on a chair in the centre of a circular stage with radius 1.5 m, facing an arc of loudspeakers positioned at approximately head height. The experiment consisted of four 20-min runs, each run containing 300 4-s trials. Trials started with four, 90-ms adapter stimuli, separated by onset-onset intervals of 100 ms. The adapter stimuli were either presented all from a loudspeaker at 0 ° (relative to straight ahead), or they alternated between loudspeakers at −30 ° (left) and +30 ° (right) or −90 ° and +90 °. A probe stimulus was then presented at 0 °, +30 ° or +90 °, being separated from the last adapter by an onset-onset interval of 600 ms. Trials in which the adapters were omitted were also included. The adapter and probe locations were selected randomly on each trial. On average, across the four runs, each condition was presented 100 times. This was a passive listening study—participants were instructed to ignore the auditory stimuli and to watch a subtitled film presented on a screen directly below the loudspeaker at 0 °.

### Stimuli

Acoustical stimuli were generated digitally with a 44.1-kHz sampling rate and 16-bit amplitude resolution, in Matlab (The Mathworks, Natick, MA), by summing pure tones with frequencies at 0.2-Hz intervals between 100 and 5000 Hz. The amplitude of each pure tone was inversely proportional to the square root of its frequency, giving equal power per octave (so-called “pink” noise); the starting phase of each tone was selected randomly from a uniform distribution. All stimuli were 90 ms in duration, including 10-ms raised cosine onset and offset ramps. When presented from one of the five loudspeakers, each stimulus had an intensity of 55 ± 1-dBA SPL at the approximate position and head height of the participants.

### Electroencephalography

EEG recordings were made using 64 Ag/AgCl electrodes, arranged according to the 5 % electrode scheme, in an elasticated cap incorporating active noise cancellation (ANT WaveGuard system, Enschede, Netherlands). Signals were referenced online to the mean across channels (average reference), with a central forehead electrode (AFz) used as ground. Signals were amplified and low-pass filtered at 500 Hz, then sampled at 1000 Hz and stored for offline processing by ASA Lab software (ANT, Enschede, Netherlands).

Recordings were processed offline using the EEGLAB toolbox (Delorme and Makeig 2004), which runs under Matlab. Much of the processing was identical to that reported by Briley et al. (2013) and Briley and Summerfield (2014). Recordings were bandpass-filtered from 0.1 to 35 Hz, split into 600-ms epochs starting 100 ms before the onset of each probe stimulus and baseline corrected to the 100-ms pre-stimulus period. Data from the four runs were then merged, and epochs containing extreme values (joint probability limits of ±3.5 SD) were removed. On average, 15.3 % of epochs were rejected, leaving 84 epochs per condition per participant. Stereotyped artefacts (eye blinks and lateral eye movements) were removed manually following statistical decomposition of the data into maximally independent components using the Infomax extended ICA algorithm (Bell and Sejnowski 1995; Lee et al. 1999) with an initial PCA decomposition that retained the ten largest principal components. Epochs were then averaged for each combination of probe location and adapter location condition.

Estimates of the aggregate neural activity as a function of time within each auditory cortex (termed “source waveforms”) were obtained from the electrode data using equivalent current dipole modelling (Scherg 1990), with one dipole at the centroid of the primary auditory area, TE1.0, in each hemisphere (Morosan et al. 2001). The orientations of the two dipoles were fitted simultaneously within a 40-ms window centred on the P2 peak of the grand-average probe response. The orientations of the dipoles were fitted, while the dipole locations were held fixed, because EEG is more sensitive to variation in dipole orientation than dipole location (Nunez and Srinivasan 2006); small differences between dipole locations and the true locations of neural generators have little effect on results. Fits were made to the P2 due to its prominence in the neural responses, though fits to the N1 gave similar (albeit noisier) results. Subsequently, for each combination of participant, cortex (left, right), adapter location condition (0 °, ±30 °, ±90 °) and probe location (0 °, +30 °, +90 °), the sizes of the N1 and P2 peaks were identified as the most negative (N1) and most positive (P2) responses in 40-ms windows centred on the time points corresponding to these peaks in the grand-average probe response. The N1 and P2 components have overlapping time courses but opposite polarities, so partially cancel; therefore, as in our previous work, we quantified neural response sizes using the peak-to-peak difference between the N1 and the P2.

### Computational Modelling

Probe response sizes, averaged across participants, were fitted with computational models based on those used by Briley et al. (2013) and Briley and Summerfield (2014). There were 18 data points available for fitting (three probe locations by three adapter locations by two auditory cortices). At the core of the models was a description of the tuning of two or three spatial channels, in the form of a tuning curve (a function relating channel output to sound-source azimuth) for each channel. Each tuning curve represents the aggregate tuning of that channel’s constituent neurons. Tuning curves were modelled using the probability density functions of generalized Gaussian distributions (Varanasi and Aazhang 1989). The generalized Gaussian has a width parameter, related to the standard deviation of a conventional Gaussian, and a shape parameter, which allows the function to adopt the conventional Gaussian shape or to have a sharper or flatter peak. Peak channel outputs were at −90 ° (left channel), 0 ° (middle channel) or +90 ° (right channel). In terms of their widths and shapes, the left and right channels were assumed to be mirror images of each other. The three channels were assumed to exist in each auditory cortex. Given the contralateral hemifield preferences of the auditory cortices (Briley et al. 2016), the peak outputs (“sizes”) of the left and right channels were allowed to differ between the cortices (the peak output represents the number, or overall activity, of neurons contributing to a channel). Specifically, for left auditory cortex, the ipsilateral (left) peak output was allowed to vary between 0 and 1, whilst the contralateral (right) peak output was fixed at 1. Likewise, for right auditory cortex, the ipsilateral (right) peak output was allowed to vary whilst the contralateral (left) peak output was fixed at 1. Allowing the peak outputs of the contralateral channels to vary instead led to peak outputs at, or close to, 1 (confirming that the ipsilateral channels are not larger than the contralateral channels). The middle channel had the same size for both cortices; this was allowed to vary between 0 and 1.

For each adapter condition, adaptation was simulated by down-scaling the tuning curve of each channel in proportion to the extent to which that channel would respond to the presented adapters. For example, in the 0 ° adapter condition, the middle channel would respond maximally to the adapters so would be maximally adapted; the left and right channels would respond more weakly, so would be more weakly adapted. The maximum amount of adaptation that could be elicited by an adapter (*a*_max_) was a further free parameter, which varied between 0 and 1. An *a*_max_ of 0.7, for example, would mean that a 0 ° adapter (which would always elicit the maximum response from the middle channel) would reduce the response of the middle channel by 70 %. Similarly, if an adapter at +30 ° elicited half the maximum response from the middle channel, then, with an *a*_max_ of 0.7, that adapter would reduce the size of the middle channel by 35 % (0.7 × 0.5). We did not model the time course of adaptation explicitly, but we did treat the 0 ° adapter as two adapters, to account for the fact that it was presented twice as often as the other adapters.

For each probe condition, response sizes were calculated by summing the adapted outputs of the channels at the probe location and multiplying the sum by a scale factor. Models were fitted to the measured probe response sizes by minimizing the sum-of-squares error (SSE) between the predicted and observed data using the genetic algorithm—a global optimization algorithm implemented in Matlab. In total, there were six free parameters for the two-channel model (left/right channel width, left/right channel shape, ipsilateral channel size for left auditory cortex, ipsilateral channel size for right auditory cortex, maximum amount of adaptation, scale factor). There were an additional three free parameters for the three-channel model (middle channel width, shape and size).

## **RESULTS**

**FIG. 1:**
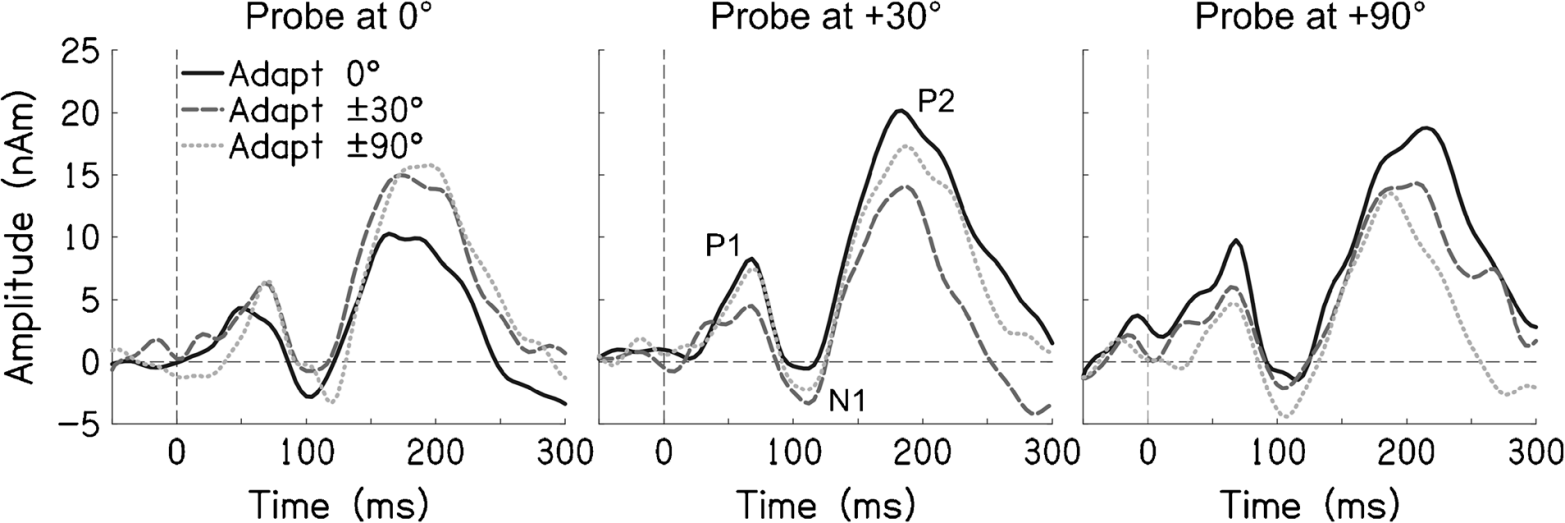
Probe responses, shown as source waveforms averaged across participants and cortices, for each combination of probe location (*panels*) and adapter locations (*lines*).

**FIG. 2:**
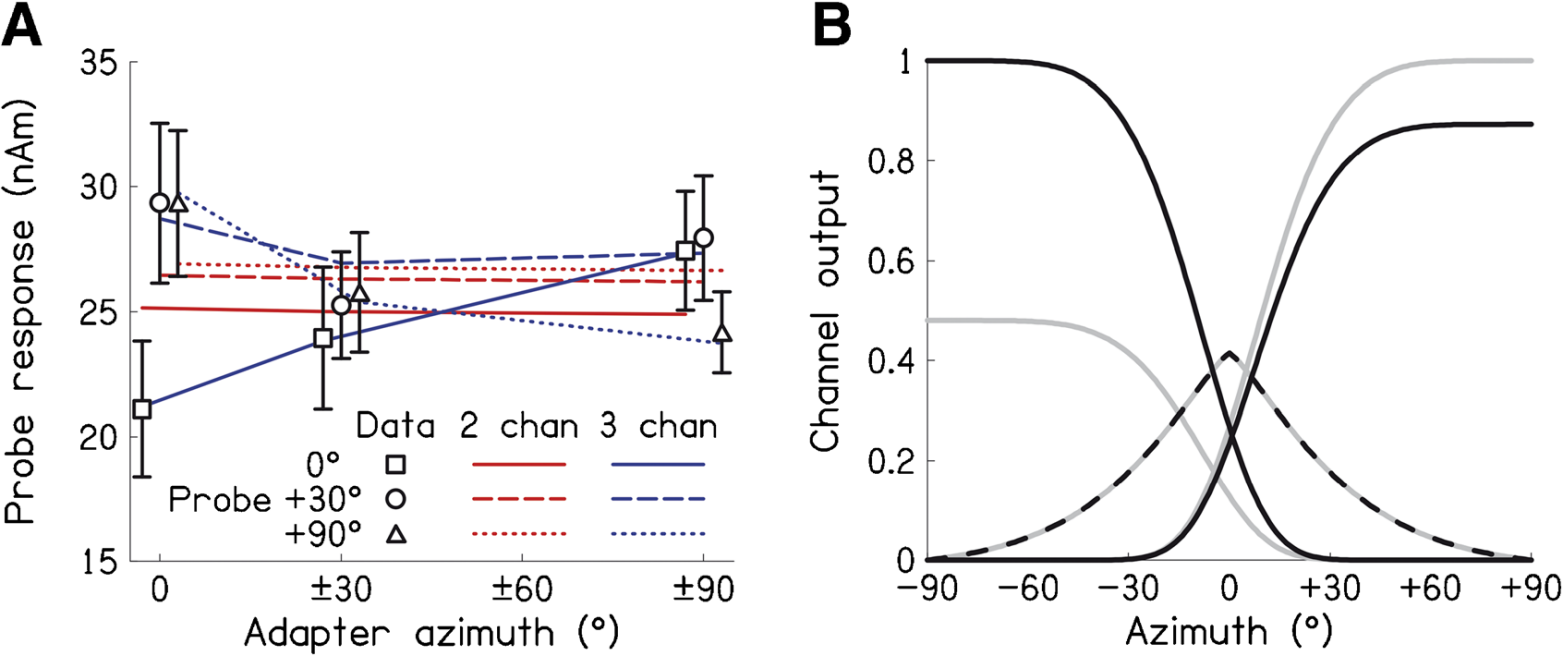
**A** Symbols show probe response size as a function of adapter locations for each probe location, averaged across cortices and participants. *Errors bars* are 95 % within-subjects confidence intervals (Morey 2008). *Red lines* show fits of a two-channel model, whilst *blue lines* show fits of a three-channel model. *Symbols* and *lines* are slightly offset horizontally for clarity. **B** Fitted channel tuning curves under the three-channel model. *Light grey*, and *black*, shows fits for left, and right, auditory cortex, respectively. Only the peak output of the ipsilateral channel was allowed to vary between cortices.

Probe stimuli elicited transient neural responses with peaks corresponding to the P1, N1 and P2 components of the auditory event-related potential (Fig. 1, responses shown collapsed across cortices; Key et al. 2005). The probe response for each condition was quantified as the peak-to-peak amplitude from the N1 to the P2, in line with our previous work (see “METHODS” section). The symbols in Figure 2A plot probe response as a function of adapter azimuth, for each of the three probe locations, collapsed across cortices. As predicted by both the two- and three-channel accounts, the response to the +90 ° probe (triangles) was smallest (i.e. adaptation was greatest) when the adapters were at ±90 °. The probe response was greatest (i.e. adaptation was least) when the adapters were at 0 °.

The two- and three-channel accounts can be distinguished by the pattern of responses when the probe location was 0 ° (squares). In this case, the two-channel account still predicts smallest responses when the adapters are at ±90 °, as these adapters will adapt the two spatial channels the most. However, the three-channel account predicts smallest responses when the adapters are at 0 °, as these adapters will adapt the midline channel the most—the channel that responds greatest to the 0 ° probe. The results support the three-channel account. Probe responses were smallest when the adapters were at 0 ° and greatest when the adapters were at ±90 °. The results for the +30 ° probe (circles) also argue against the two-channel account, since adaptation was not greatest with the ±90 ° adapters.

We conducted a linear mixed model analysis of the probe response sizes, with probe location (0 °, +30 °, +90 °), adapter location (0 °, ±30 °, ±90 °) and auditory cortex (left, right) as fixed factors and participants as a random factor. The three-way interaction was non-significant [*F*(4, 255) = 0.687, *p* = 0.601], so was removed from the model. The main effect of probe location was significant [*F*(2, 259) = 5.871, *p* = 0.003], with slightly smaller responses, on average, to the 0 ° probe than the +30 ° (*p* = 0.001) or +90 ° (*p* = 0.024) probes. The interaction of probe location and auditory cortex was significant [*F*(2, 259) = 7.304, *p* = 0.001]. The interaction arose because response sizes differed overall between probe locations in left auditory cortex [*F*(2, 259) = 11.587, *p* < 0.001], but not right auditory cortex [*F*(2, 259) = 1.588, *p* = 0.206]. In left auditory cortex, responses were 25 and 27 % larger to the +30 ° and +90 ° probes, respectively, than the 0 ° probe (both *p* < 0.001). This result suggests a preference for right hemifield locations in left auditory cortex, which is not apparent in right auditory cortex. Preference for left hemifield locations in right auditory cortex cannot be properly assessed as probe stimuli were not presented in the left hemifield. Neither of the remaining main effects was significant [adapter location: *F*(2, 259) = 1.645, *p* = 0.195; auditory cortex: *F*(1, 259) = 1.647, *p* = 0.201], nor was the two-way interaction of adapter location and auditory cortex [*F*(2, 259) = 1.268, *p* = 0.283].

In seeking evidence for a midline spatial channel, the critical analysis is the interaction of probe location and adapter location. This interaction was highly significant [*F*(4, 259) = 6.318, *p* < 0.001]. There was a significant effect of adapter location for the +90 ° probe [*F*(2, 259) = 4.678, *p* = 0.010], with larger responses following the 0 ° adapters than the ±90 ° (*p* = 0.003) or ±30 ° (*p* = 0.040) adapters, in line with both the two- and three-channel accounts. The effect of adapter location was also significant for the 0 ° probe [*F*(2, 259) = 6.724, *p* = 0.001], with larger responses following the ±90 ° adapters than the 0 ° (*p* < 0.001) or ±30 ° (*p* = 0.044) adapters, in line with the three-channel, but not the two-channel, account. The effect of adapter location for the +30 ° probe only approached significance [*F*(2, 259) = 2.880, *p* = 0.058]; the response was larger following the 0 °, than ±30 °, adapters (*p* = 0.019).

### Computational Modelling

Two- and three-channel computational models, based on those developed by Briley et al. (2013) and Briley and Summerfield (2014), were fitted to the probe response sizes averaged across participants (three probe locations by three adapter locations by two auditory cortices, giving 18 data points). As described in the “METHODS” section, the fitting procedure adjusted model parameters describing the left-, right- and (for the three-channel model) middle-channel tuning curves, as well as a parameter determining the size of the adaptation effect and a parameter determining the overall scaling of response sizes, in order to minimize the sum-of-squares error (SSE) between the measured probe response sizes and those predicted by the model.

Figure 2A shows the fits of the two- and three-channel models as lines (red and blue, respectively) alongside the data as symbols. The two-channel model was unable to account for the detailed pattern of response sizes across the different adapter and probe locations. Instead, it only captured the mean response to each probe location and showed a slight downward trend with increasing adapter magnitude. The *R*^2^ goodness of fit was 0.61. The SSE was 115.32, giving a root-mean-squared error (RMSE) of 2.53 nAm. Given the poor two-channel fit, estimates of channel tuning from this model are meaningless. On the other hand, the three-channel fit showed variations of response size as a function of adapter location that were consistent with the patterns in the observed data for each probe location. Specifically, the response to the +90 ° probe (data: triangles, model: dotted blue line) was smallest with the ±90 ° adapters, whilst the response to the 0 ° probe (data: squares, model: solid blue line) was smallest with the 0 ° adapters. The model also captures some of the (non-significant) dip in response size for the +30 ° probe with the ±30 ° adapters. The *R*^2^ goodness of fit was 0.93, meaning that the fit explained 93 % of the variance about the mean in the observed data. The RMSE was thus low, at 1.20 nAm (SSE of 25.69). An *F*-test, which compares the reduction in SSE to the reduction in degrees of freedom when moving from the less complex two-channel model (six free parameters) to the more complex three-channel model (nine free parameters), indicated that the 78 % reduction in SSE was highly significant [*F*(3, 9) = 10.469, *p* = 0.003].

he fitted channel tuning curves for the three-channel model are shown in Figure 2B. As described in the “METHODS” section, to account for the reported contralateral hemifield preferences of left and right auditory cortex (see Briley et al. 2016, for a discussion), the peak output of the ipsilateral channel was allowed to vary between 0 and 1, separately for each cortex (the peak output of the contralateral channel was set to 1 in each case). All other channel parameters, including the left and right channel widths and shapes, and the parameters governing the middle channel were the same across the cortices. For left auditory cortex (light grey lines in Fig. 2B), the fit set the ipsilateral (left) channel peak output to 0.48, thus implying that the size of the ipsilateral channel was 48 % of the size of the contralateral channel. For right auditory cortex (black lines), the contralateral preference was less marked, with the ipsilateral (right) channel size set at 87 % of the size of the contralateral channel. The middle channel peak output was set to 0.41, a bit under half that of the largest channel in each cortex. The left and right channels were broader than the middle channel; the full width at half maximum (FWHM) of the left/right channels was 160 °, whilst the FWHM of the middle channel was 51 °. Fitted parameters for the two- and three-channel models are given in Table 1.

**TABLE 1:** Fitted parameter values for the three-channel model

Ipsilateral (left) channel size in left auditory cortex	0.480
Ipsilateral (right) channel size in right auditory cortex	0.874
Middle channel size	0.415
Left/right channel width (°)	60.5
Middle channel width (°)	25.3
Left/right channel shape	5.43
Middle channel shape	1.26
Maximum amount of adaptation	0.342
Scale factor (nAm)	38.5

## **DISCUSSION**

For a probe stimulus presented in peripheral space (+90 ° azimuth), adaptation was greatest when the adapter stimuli were also presented in peripheral space (±90 °) and least when adapter stimuli were presented at the midline (0 °). This result is consistent with both the two-channel and three-channel accounts. However, in line with the three-channel account only, when a probe stimulus was presented at the midline, adaptation was greatest when adapter stimuli were also at the midline and least when adapter stimuli were at the periphery. Thus, neural responses from auditory cortex, recorded under passive listening conditions, support the results of active listening, psychophysical studies (Dingle et al. 2010, 2012) in arguing for the existence, in humans, of a midline-tuned spatial channel alongside the previously hypothesized left- and right-tuned channels.

### Evidence for a Midline Spatial Channel from Human Studies

Previous human physiological studies have presented evidence for a two-channel account of spatial representation in auditory cortex using EEG (Magezi and Krumbholz 2010; Briley et al. 2013; Briley and Summerfield 2014) or its magnetic counterpart MEG (Salminen et al. 2009, 2010) and using functional magnetic resonance imaging (fMRI) (Stecker et al. 2015; Trapeau and Schönwiesner 2015; McLaughlin et al. 2016). However, these studies were not designed to contrast the two- and three-channel accounts. For example, fMRI studies have measured responses in voxels aligned with regions of auditory cortex to stimuli at different azimuths and have generally found larger responses to more lateral azimuths, especially towards the side contralateral to the cortex being measured (Stecker et al. 2015; Trapeau and Schönwiesner 2015; McLaughlin et al. 2016). Whilst this outcome provides support for a two-channel account, the measured response in each voxel is still the average of the responses of many thousands of neurons. Given the suspected greater number of left/right-tuned, than midline-tuned, neurons, it is likely that voxel responses will be dominated by left/right-tuned neurons (neurons with different location tuning curves appear intermixed within auditory cortex, so there will not be voxels recording from neurons with only midline tuning). A secondary peak in voxel responses at the midline would not necessarily be expected if the middle channel were smaller than the left/right channels. Rather, voxel responses would reflect a summation of the tuning curves of neurons within an area/voxel (such a summation of the tuning curves in Fig. 2B, for example, does not give a peak at the midline). Of course, EEG, used in the current study, averages from even greater numbers of neurons than fMRI. It is the use of an adaptation paradigm, with appropriate comparisons (effects of midline and lateral adapters on responses to midline and lateral probes), which enabled the current study to separate out the contribution of the middle channel. Adaptation is frequently used to study neural populations beyond the typical resolution of a neuroimaging modality (Grill-Spector and Malach 2001).

Salminen et al. (2009, 2010), Magezi and Krumbholz (2010) and Briley et al. (2013) obtained evidence for the two-channel model using an adaptation paradigm with EEG or MEG. However, Salminen et al. examined the effect of adapter location on responses to a probe stimulus at −45 ° or a probe presented with an interaural time difference (ITD, one of the auditory cues to sound-source location) of −0.4 ms, which would also be perceived at an intermediate location. Both probe stimuli would only elicit weak responses from the middle channel. Magezi and Krumbholz (2010) presented pairs of stimuli, back-to-back, that differed in ITD. They included inward transitions—from lateral locations towards the midline—and outward transitions—from the midline, laterally. They found that outward transitions elicited larger responses than inward transitions, consistent with a two-channel model since outward transitions move from a low point in the left or right channel tuning curve to a high point. However, inward transitions still elicited substantial neural responses. This is consistent with the presence of a midline-tuned channel that is smaller than the left/right channels, which is compatible with the evidence in Figure 2B.

Briley et al. (2013) replicated Magezi and Krumbholz’ result using stimuli presented from an array of loudspeakers, thus incorporating the full range of cues to sound-source location, and demonstrated that a two-channel model could account reasonably well for neural responses to stimuli that moved between −60 °, −30 °, 0 °, +30 ° and +60 °. However, responses to stimuli at 0 ° were somewhat larger than predicted by the model, an effect that could be explained by an additional contribution to the response from a midline-tuned channel. Briley et al. demonstrated that a two-channel model outperformed a model incorporating many, narrowly tuned channels spaced at 1 ° intervals across the frontal plane, but they did not examine the case of three broadly tuned channels like those modelled in the present study.

### Evidence for a Midline Spatial Channel from Animal Studies

Whilst few neurons in mammalian auditory cortex respond maximally to midline locations under anaesthesia (Stecker et al. 2003, 2005a, b), substantial numbers respond under awake conditions (Lee and Middlebrooks 2011, 2013; Zhou and Wang 2012). Zhou and Wang (2012) found that approximately 50 % of location-sensitive neurons in primary auditory cortex and adjacent fields of marmoset monkeys responded maximally to the contralateral hemispace, whilst roughly 25 % each responded maximally to the midline and the ipsilateral hemispace. Our results provide support for the existence of a midline spatial channel in humans. Preliminary computational modelling suggests that this channel is smaller (e.g. has fewer, or has less active, contributing neurons), and narrower, than the left- and right-tuned channels (Fig. 2B). This pattern is consistent with the aggregate tuning of neurons classed as left/right or midline tuned reported by Zhou and Wang (their Fig. 8). Note that the left/right channel tuning curves measured by Zhou and Wang change rapidly near the midline but are relatively flat at greater absolute azimuths; those properties are exhibited by our fitted tuning curves (and the average voxel tuning curves measured with human fMRI by Trapeau and Schönwiesner 2015, their Fig. 8C). It is possible that broader tuning curves in earlier studies (see Stecker et al. 2005a, b) reflect the use of anaesthesia. In awake animals, Lee and Middlebrooks (2011, 2013) found increases in the sharpness of location tuning during relatively non-specific attention to auditory stimuli; a similar effect might account for differences in tuning under anaesthesia and without anaesthesia, especially given the greater prominence of midline-tuned neurons without anaesthesia.

A further issue to consider is whether there are additional, perhaps smaller, spatial channels beyond the three suggested by the current study. Indeed, the possibility of additional channels was acknowledged by Stecker et al. (2005a, b) in their study on two-channel representation in cat auditory cortex, referring to earlier work by Wise and Irvine (1985) that identified three subpopulations of neurons tuned to ILDs (interaural level differences, another of the auditory cues to sound location) in each superior colliculus of cats. The largest population responded maximally to ILDs indicating far contralateral locations, the second largest to ILDs indicating near-midline locations and the smallest to intermediate ILDs. In the current study, the possibility of channels intermediate between the left/right channels and the midline channel is raised by the (non-significant) dip in response to the +30 ° probe following the ±30 ° adapters (Fig. 2A); our computational model can produce a dip here without an additional channel, but the dip is probably not large enough to match the data. To clearly identify additional, smaller, channels in humans, it would be necessary to conduct experiments with a wider range of adapter and probe locations. Neurons with more complex, multimodal tuning curves may also need incorporating into a comprehensive model of sound localization. In principle, sufficient-sized groups of neurons with similar, complex, tuning curves could be probed with an adaptation paradigm, although guidance as to the shapes of these curves would be required from single-unit studies with animals.

Starting from the assumption that the ITDs commonly occurring in a species’ environment should be coded with maximal accuracy, Harper and McAlpine (2004) and Harper et al. (2014) suggested that different species may represent ITDs differently, using two channels, three channels or, indeed, many narrowly tuned channels. Moreover, if the representation of ITDs is allowed to differ across frequency within a given species, then their work suggests that the presence of two, three or more channels may depend on sound frequency. In the current study, we used a broadband stimulus, spanning a wide frequency range (100–5000 Hz); it is possible, therefore, that we are seeing the responses of a mixture of channels sensitive to different sound frequencies. Future work should use narrowband stimuli to seek evidence for different numbers of channels in different frequency ranges.

### Summary

We report physiological evidence for a midline-tuned spatial channel in human auditory cortex in addition to channels tuned broadly to left and right auditory space. Our evidence supports psychophysical evidence reported by Dingle et al. (2010, 2012) and is in line with recent physiological studies on awake mammals (Lee and Middlebrooks 2011, 2013; Zhou and Wang 2012). Key issues for future research include whether there are additional channels beyond the three identified here, and how information from multiple spatial channels is combined to provide a single estimate of source-source location. The latter issue is particularly important, since we have shown that reasonable predictions of human spatial acuity can be made under the assumption of left and right channels alone (Briley et al. 2013; Briley and Summerfield 2014). A third channel may make location estimates more robust to sound-level changes, it may assist in complex listening environments, or it may serve an alerting role, identifying when objects are approaching the midline and thus potentially becoming more important. That the midline channel contributes to sound localization is supported by the psychophysical effects of Dingle et al. Note that, in opponent-process models such as the two/three-channel models, each channel provides greatest acuity for regions of space falling within the steepest portions of the channel’s tuning curve (McAlpine et al. 2001; Stecker et al. 2005a). This means that the left/right channels enable high spatial acuity near the midline. A middle channel, then, would particularly support spatial acuity in the mid-periphery, where the rate of change of its tuning curve is steepest. Finally, the greater prominence of the middle channel (relative to the left and right channels) in animal studies under awake, than anaesthetized, conditions suggests a functional difference between the channels that warrants further exploration.
